# Clinical Outcomes in a Large Canadian Centralized CLL Clinic Based on Treatment and Molecular Factors over a Decade

**DOI:** 10.3390/curroncol30070472

**Published:** 2023-07-05

**Authors:** Jiayu Yang, Lin Yang, Bryan Tordon, Oliver Bucher, Zoann Nugent, Ivan Landego, Nicole Bourrier, Kelsey Uminski, Kevin Brown, Mandy Squires, Aaron J. Marshall, Sachin Katyal, Salah Mahmud, Kathleen Decker, Marc Geirnaert, David E. Dawe, Spencer B. Gibson, James B. Johnston, Versha Banerji

**Affiliations:** 1 Department of Internal Medicine, Rady Faculty of Health Sciences, University of Manitoba, Winnipeg, MB R3E 3P4, Canadajjohnsto@cancercare.mb.ca (J.B.J.); 2 Department of Epidemiology, CancerCare Manitoba, Winnipeg, MB R3E 0V9, Canadakdecker@cancercare.mb.ca (K.D.); 3 Department of Biochemistry and Medical Genetics Max Rady College of Medicine, Rady Faculty of Health Sciences, University of Manitoba, Winnipeg, MB R3E 3P4, Canada; zoann.nugent@umanitoba.ca (Z.N.); spencer.gibson@umanitoba.ca (S.B.G.); 4 Paul Albrechtsen Research Institute CancerCare Manitoba, Winnipeg, MB R3E 0V9, Canadasachin.katyal@umanitoba.ca (S.K.); 5 Department of Immunology, Max Rady College of Medicine, University of Manitoba, Rady Faculty of Health Sciences, Winnipeg, MB R3E 0T5, Canada; aaron.marshall@umanitoba.ca; 6 Department of Pharmacology and Therapeutics, Max Rady College of Medicine, Rady Faculty of Health Sciences, University of Manitoba, Winnipeg, MB R3E 3P4, Canada; 7 Department of Community Health Sciences, Max Rady College of Medicine Community Health Sciences, Winnipeg, MB R3E 0W2, Canada; salah.mahmud@umanitoba.ca; 8 Department of Pharmacy, CancerCare Manitoba, Winnipeg, MB R3E 0V9, Canada; mgeirnaert@cancercare.mb.ca

**Keywords:** real world evidence, molecular testing, CLLIPI, treatments, outcomes

## Abstract

FISH cytogenetics, TP53 sequencing, and IGHV mutational status are increasingly used as prognostic and predictive markers in chronic lymphocytic leukemia (CLL), particularly as components of the CLL International Prognostic Index (CLL-IPI) and in directing therapy with novel agents. However, testing outside of clinical trials is not routinely available in Canada. As a centralized CLL clinic at CancerCare Manitoba, we are the first Canadian province to evaluate clinical outcomes and survivorship over a long period of time, incorporating the impact of molecular testing and the CLL-IPI score. We performed a retrospective analysis on 1315 patients diagnosed between 1960 and 2018, followed over a 12-year period, where 411 patients had molecular testing and 233 patients had a known CLL-IPI score at the time of treatment. Overall, 40.3% (*n* = 530) of patients received treatment, and 47.5% (*n* = 252) of patients received multiple lines of therapy. High-risk FISH and CLL-IPI (4-10) were associated with higher mortality (HR 2.03, *p* = 0.001; HR 2.64, *p* = 0.002), consistent with other studies. Over time, there was an increase in the use of targeted agents in treated patients. The use of Bruton’s tyrosine kinase inhibitors improved survival in patients with unmutated IGHV and/or TP53 aberrations (HR 2.20, *p* = 0.001). The major cause of death in patients who received treatment was treatment/disease-related (32%, *n* = 42) and secondary malignancies (57%, *n* = 53) in those who were treatment-naïve. Our data demonstrate the importance of molecular testing in determining survivorship in CLL and underpinning the likely immune differences in outcomes for those treated for CLL.

## 1. Introduction

Chronic lymphocytic leukemia (CLL) is a clonal lymphoproliferative disorder of abnormal B-lymphocytes and remains the most common leukemia in older adults in North America [1,2,3,4,5]. Canadian statistical data (excluding Quebec) showed an estimated incidence rate of 5.6 per 100,000 people [6], with 1725 new cases (2018) and deaths in 2020 [7]. Local data suggests that the true incidence of CLL is higher, depending on referral practices, access to flow cytometry test results, and cancer registries [1]. Small lymphocytic lymphoma (SLL) is managed similarly to CLL but requires less than 5 × 10^9^/L peripheral clonal B lymphocytes in the presence of organomegaly or lymphadenopathy [8]. For simplicity, we will use CLL to represent both CLL and SLL patients in our cohort. While many patients have an indolent course at the time of diagnosis, other patients have more aggressive disease and require treatment, sometimes with multiple relapses. Over the last decade, the treatment landscape for CLL has rapidly evolved. In addition to the traditional clinical parameters of patient age, functional status, and Rai stage, molecular markers including loss of chromosome 17p, TP53 mutation testing, and immunoglobulin heavy chain variable region (IGHV) mutational status have identified patients who would benefit from immune targeted therapies [8,9,10]. Using our longitudinal study cohort of over 12 years, we were able to capture the demographics and clinical outcomes of individuals treated in the pre-immune and immune eras. To our knowledge, this is the first Canadian study to report outcomes based on a large patient cohort integrating the prognostic significance of the CLL International Prognostic Index (CLL-IPI) score.

Variability in outcomes is now known to be in part related to disease-associated genetic heterogeneity that may be detected through laboratory testing [11]. Cytogenetic abnormalities detected through fluorescence in-situ hybridization (FISH) are associated with either a better or worse prognosis [11,12,13,14,15,16]. In the original study of FISH subgroups by Dohner et al., ~80% of patients had at least one of the now commonly recognized chromosomal abnormalities: del(13q), trisomy 12, del(11q), and del(17p). Del(13q) was associated with a more favourable prognosis; a normal karyotype or trisomy 12 is considered intermediate risk; and either del(11q) or del(17p) had the worst outcomes [13]. While del(17p) results in a defective tumour-suppressor protein p53 (TP53) gene, independent mutations affecting TP53 have also been identified as having similar poor outcomes [11,14,17,18]. Results of FISH testing and TP53 mutations change over time and with exposure to chemoimmunotherapy agents [16].

Similarly, the mutational status of the IGHV is associated with disease prognosis. Unmutated IGHV is associated with worse outcomes, including overall survival as well as resistance to standard chemotherapy agents [19,20,21]. While previously reserved for research purposes, IGHV mutational status is now available through clinically accredited labs for treatment decision-making. In contrast to FISH and TP53 mutational analysis, IGHV mutational status does not change over time or with exposure to chemoimmunotherapy [22]. There are, however, subsets that may do worse despite being labelled dichotomously, such as subset 2 [9,10,23]. In addition, as complex karyotypes are not measured locally, we did not address them in this manuscript, but they also play a role in the prediction of poor outcomes and response to therapy [24].

The Rai and Binet clinical staging systems are methods of prognostication that incorporate physical findings on exam and laboratory data to partake in treatment decisions and predict outcomes in patients with CLL [25,26,27]. With the advent of advanced laboratory tests that offer further prognostic information, the international workshop on CLL (iwCLL) guidelines were revised in 2018 and now include routine testing of FISH, TP53 sequencing, and IGHV in patients who are being considered for treatment [28]. It is our standard practice in the publicly funded system to use molecular testing during treatment as opposed to at the time of diagnosis [4]. Since 2015, Bruton’s tyrosine kinase (BTK) inhibitor, ibrutinib, has been introduced and has significantly changed the treatment landscape for CLL. At that time, the Canadian guidelines in the front-line setting recommended ibrutinib for individuals with del(17p) or TP53 mutations or IGHV unmutated status as opposed to other chemoimmunotherapy agents [4]. In fit patients without del(17p) or TP53 mutation and mutated IGHV status, either fludarabine-cyclophosphamide-rituximab (FCR) or bendamustine-rituximab (BR) are recommended depending on fitness [4,29,30]. Ibrutinib is also available if the patient is deemed a suitable candidate. Otherwise, unfit older patients without high-risk mutations are recommended to receive chlorambucil-obinutuzumab with ibrutinib or venetoclax, reserved for those who are intolerant [4,31,32,33,34]. Acalabrutinib [35] was not available unless on a clinical trial during this study, nor was the combination of venetoclax and Obinutuzumab [36]. In the relapsed setting, ibrutinib [37] or venetoclax [38,39] were both available as monotherapy (outside of Quebec), and venetoclax was usually sequentially administered after ibrutinib intolerance/failure or progression [39,40]. Venetoclax in combination with anti-CD20 antibodies (in the frontline or relapsed setting) was not available at the time of this study.

To risk stratify patients, the CLL-IPI was developed. The score utilizes both clinical and laboratory characteristics to stratify patients [41,42,43]. The factors of the CLL-IPI associated with worse outcomes include age >65 years, Rai stages I–IV, presence of del(17p) or mutated TP53, unmutated IGHV status, and serum beta-2 microglobulin > 3.5 mg/L [43]. Based on the individual patient characteristics, a risk category of low (0–1), intermediate (2–3), high (4–6), and very high (7–10) is generated (with individual overall survival being 93%, 79%, 63%, and 23% over 5 years, respectively). The CLL-IPI has been externally validated in patients with untreated CLL with similar outcomes [42,43] and is now being used in clinical trials to understand the impact of risk on early treatment in the era of novel agents (EVOLVE: S1925 NCT04269902).

Population-based data can be difficult to track across centres where the referral base is broad and practice patterns may differ between locations. In addition, most of the data acquired reflects an inherent bias towards high-risk populations, as those patients are more likely to be referred to a tertiary care centre. Manitoba is unique, with a population-based cohort and a centralized intake system to evaluate testing, changes to drug therapy, and outcomes over time [1]. For these reasons, our research group is uniquely positioned to evaluate the changing landscape of molecular testing in CLL and its effects on treatment patterns. Our objectives are to evaluate CLL/SLL patients seen in our centralized clinic for the number of FISH and IGHV mutational status tests performed, evaluate the distribution of results in our tested cohort, and establish overall survival (OS) by prognostic category and CLL-IPI scores. In parallel, we assessed changes in treatment regimens used over time, in the pre- and post-molecular testing eras, to correlate with outcomes, survival, and causes of death.

## 2. Methods

This retrospective study was approved by the University of Manitoba Research Ethics Board HS20746 (H2017:140).

### 2.1. Study Population

We performed a retrospective analysis of individuals with MBL/CLL/SLL seen in the centralized CLL clinic in Manitoba between January 2006 and December 2018. Due to inability to accurately capture those who progressed to CLL from MBL, all patients initially diagnosed with MBL/CLL and SLL were included in the analysis. Thus, diagnosis of MBL/CLL or SLL in our study is defined at the time of confirmatory peripheral blood flow cytometry or tissue pathology, respectively, rather than at time of treatment. We utilized the CLL CAISIS database, which incorporates clinical and laboratory information from patient records and our electronic medical record, ARIA, as well as the CancerCare Manitoba pharmacy database. We evaluated patient data, including demographics, time of diagnosis, date of death if applicable, and cause of death where available. FISH, TP53 testing, and IGHV mutational status data were collected at time of treatment, where available. Patients were analyzed based on recognized prognostic subgroups and CLL-IPI with regards to overall survival, number of CLL directed therapies, and time to next treatment (TTNT). CLL-IPI was calculated at the time treatment was indicated based on age, beta2-microglubulin, Rai stage, TP53 mutation, and IGHV mutational status. For patients that received treatment, number of lines of therapy were collected and utilized. Causes of death were obtained from clinical chart and registry when available.

### 2.2. Mutational Studies

We utilized a combination of pre-existing interphase FISH cytogenetics results performed through send-out testing to Mayo Clinic and those performed locally to evaluate for cytogenetic abnormalities. Thresholds for cut-off values for positive results were based on Clinical Laboratory Improvement Amendments (CLIA)-approved standard of the reports. Individuals were classified as follows: del(13q) were low risk, normal cytogenetics or trisomy 12 were intermediate risk, and del(11q) or del(17p) were high risk. Although del(11q) is no longer considered high-risk, we classified it as such given the era of our analysis. Ranking was hierarchical. If multiple abnormalities were present, patients were placed in the highest applicable risk category. If multiple FISH tests were available, only the first was included in the interpretation.

IGHV mutational status testing was performed via send-out to the Mayo Clinic Laboratories during the study period or obtained from the Manitoba blood and marrow bank. After March 2018, this was performed routinely when treatment was indicated for patients in Manitoba via a clinically approved laboratory at the Mayo Clinic. Patients who did not have either FISH or IGHV mutational status testing available were excluded from final analysis of overall survival for these outcome analyses but were included in the analysis of the entire cohort. TP53 mutation testing was included where available. CLL-IPI score was determined where possible, with risk categories ranging from low to very high.

### 2.3. Survival Studies

Descriptive statistics were analyzed using the Statistical Package for the Social Sciences (SPSS Statistics 29.0 for Windows; SPSS Inc., Chicago, IL, USA). Patients were grouped by whether they received any treatment and by disease risk status, including IGHV mutation status, FISH cytogenetics, and CLL-IPI score. Overall survival (OS) was defined as the time from disease diagnosis to the time of death or end of study. Time to next treatment was defined as the time from start of one line of therapy to the start of the next line of therapy. Kaplan-Meier curves were used to describe the relationship between FISH risk category, mutational status, CLL-IPI score, and OS. Log-rank test was used to determine survival differences using the Kaplan-Meier method. The relationships between CLL treatment, FISH, IGHV, and CLL-IPI with survival were each assessed using univariable Cox proportional hazards modelling. We pursued a descriptive assessment of overall survival from time of starting each line of CLL treatment, where the same patients may be represented in multiple groups (e.g., those who received 2nd line treatment are also included in the group that received 1st line treatment). Chi-square test and Fisher Exact tests were used to check for significance in differences for descriptive statistics. *p*-value ≤ 0.05 was used as a cut-off for significance. 

All authors had access to the primary data. VB, JBJ, SM, SBG, and AM received funding. VB and JBJ conceived the study. BT, VB, and JBJ drafted original Manuscript. ZN, JY, and LY performed statistical analysis and review. JY, LY, DD, and VB revised the Manuscript. KD and OB provided statistical guidance to ZN.

## 3. Results

### 3.1. Baseline Demographics

Between 2006 and 2018, 1315 patients were seen in the centralized Manitoba CLL clinic (Table 1). In total, of 791 (60.2%) were male and 524 (39.8%) were female. Two hundred patients (15.2%) had MBL, 215 (16.3%) had SLL, and 900 (68.4%) had CLL at diagnosis. The median age was 67.4 years for males and 68.6 years for females. The median age at the time of treatment was 69.8 for males and 70.3 for females.

For CLL patients, the Rai stage was calculated for 900 patients at diagnosis. The most common stages were Rai 0 (51.7%, *n* = 465) and Rai 1 (24.2%, *n* = 218). At diagnosis, few patients are in an advanced stage, with 29 (3.2%) patients in stage 3 and 21 (2.3%) in stage 4. Of the total cohort, 530 (40.3%) patients underwent treatment. In total, 785 (59.7%) patients did not receive (Table 1) or require treatment for CLL prior to either death (24.1%, *n* = 189) or the end of the study period (75.9%, *n* = 596).

### 3.2. Molecular Profiles

FISH cytogenetic testing was available for 411 patients during the study period. Del(13q) was present in 187 (45.5%), trisomy 12 was present in 94 (22.9%), del(11q) in 65 (15.8%), del(17p) in 30 (7.3%), and 96 (23.4%) had normal cytogenetics. IGHV mutational status testing was performed on 835 patients; 353 (42.3%) were unmutated and 482 (57.7%) were mutated. Of these patients, 66.8% (*n* = 322) patients with mutated IGHV and 32.6% (*n* = 115) patients with unmutated IGHV did not require treatment for their CLL. The CLL-IPI was calculated for 233 patients, with 60 (25.8%) in the low-risk category, 100 (42.9%) in the intermediate-risk category, 62 (26.6%) in the high-risk category, and 11 (4.7%) in the very high-risk category (Table 1).

### 3.3. Overall Survival

**Figure 1:**
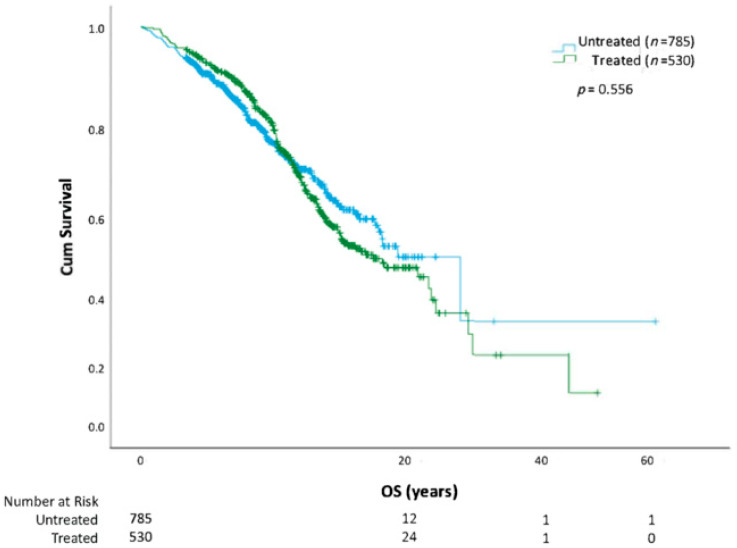
Time to death and overall survival (OS) stratified by treatment. Overall survival stratified by treatment status.

**Figure 2:**
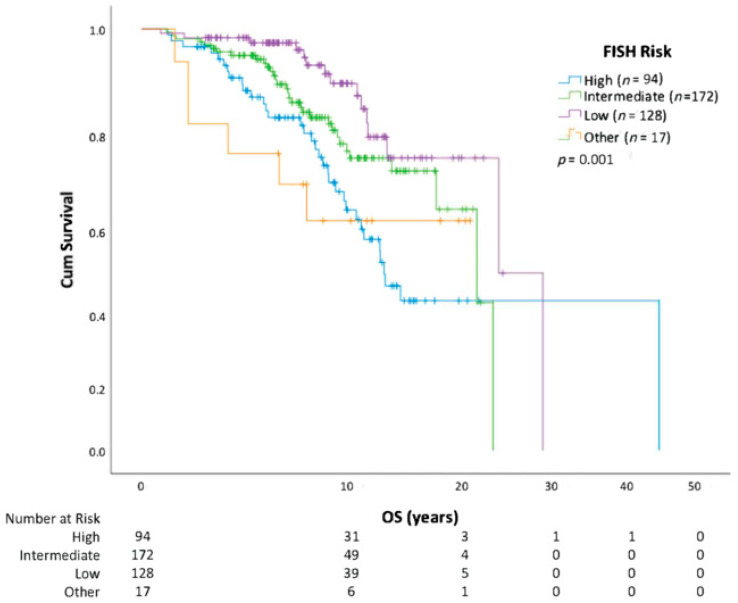
Overall survival (OS) stratified by FISH cytogenetics risk category. “Other” includes del(6q) and duplicate 13q. Del(11q) included in high risk.

**Figure 3:**
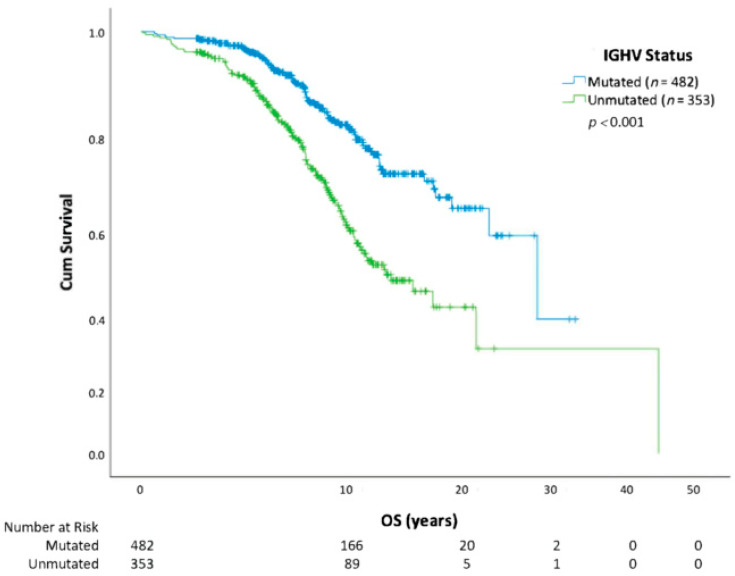
Overall survival (OS) stratified by IGHV mutational status. IGHV, immunoglobulin heavy chain variable region gene.

**Figure 4:**
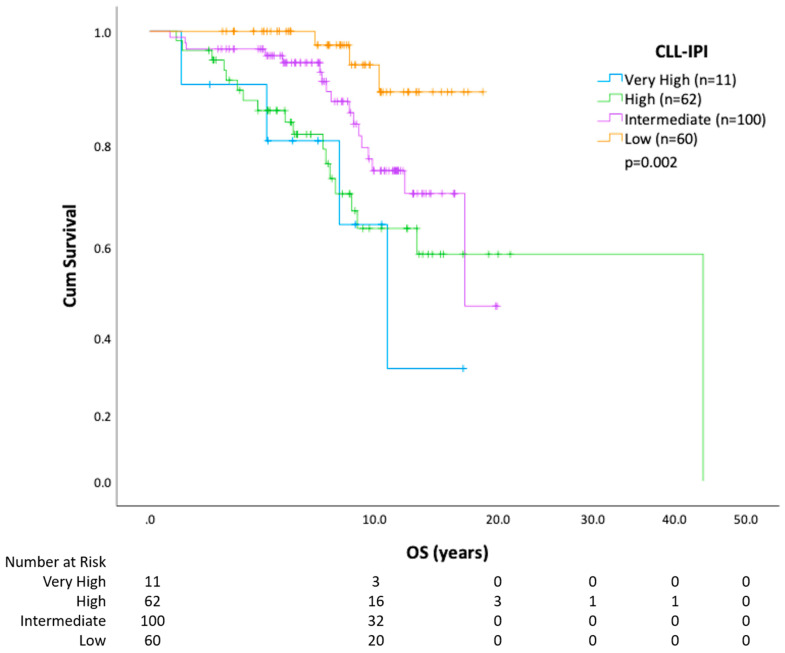
Overall survival (OS) stratified by CLL-IPI risk category. CLL-IPI, Chronic Lymphocytic Leukemia-International Prognostic Index.

**Figure 5:**
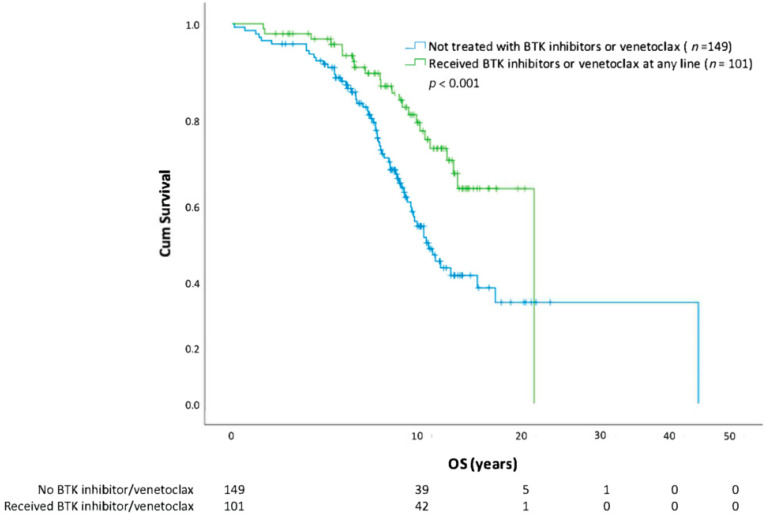
Overall survival (OS) of patients with unmutated IGHV and/or TP53 aberration stratified by treatment with BTK inhibitors (*n* = 98) or venetoclax (*n* = 3) at any line of therapy.

Using Kaplan-Meier methods, we show that overall survival in those who receive CLL treatment versus those who do not is similar (Figure 1). When the analysis was repeated using the first treatment as a starting point, receiving a second treatment also negatively affected OS (HR = 2.30; CI 1.55–3.40; *p* < 0.0001) compared with patients who only required one line of therapy. A descriptive assessment of median survival from the start of each line of therapy is outlined in Table 2. Survival analysis (from date of diagnosis to death) by CLL risk category with FISH cytogenetics, IGHV, and CLL-IPI is further outlined (Figure 2, Figure 3 and Figure 4). In all patients, regardless of treatment, when evaluating overall survival by molecular risk, patients with high-risk FISH cytogenetics classification had a shorter survival (HR 2.03, CI 1.31–3.14, *p* = 0.001) compared with low and intermediate FISH risk patients. In the same cohort, we observed worse OS in patients with unmutated IGHV (HR 2.32, CI 1.74–3.09, *p* < 0.001). Patients with high-risk CLL-IPI (4–10) also had a shorter OS than patients in lower-risk categories (HR 2.64, 95% CI 1.43–4.89, *p* = 0.002). For patients with unmutated IGHV and/or TP53 aberrations defined as TP53 mutation or del(17p), treatment with BTK inhibitors (*n* = 98) or venetoclax (*n* = 3) improved survival (HR 2.20, *p* = 0.001) (Figure 5).

### 3.4. Time to Treatment and Treatment Types

Progression-free survival is difficult to measure outside of a clinical trial due to the lack of routine imaging in the management of CLL [44,45,46]. TTNT is often used as a measure to capture progression and the need for treatment as a clinically meaningful endpoint in CLL [44,45,46]. In our cohort, 530 patients were treated, with a time to first treatment ranging from 0 to 469 months. Of the treated patients, 252 (47.5%) received a second line of therapy with a TTNT ranging from 0 to 547 months, and 126 (23.8%) patients went on to a third line of treatment with a TTNT of 0-137 months. In total, 67 (12.6%) of patients received four lines or more of therapy (Table 3 and Table 4).

**Figure 6:**
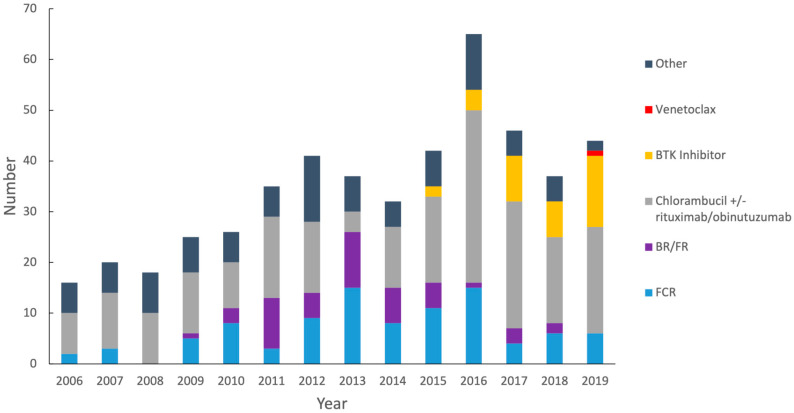
Types of therapy by year of front-line therapy initiation. (A) Number of each regimen given by year of front-line therapy initiation. “Other” includes fludarabine, fludarabine/prednisone, fludarabine, cyclophosphasphide, fludarabine/busulfan/methotrexate, cyclophosphamide/prednisone, cyclophosphamide/dexamethasone, cyclophosphamide/vincristine/prednisone, bendamustine, cyclophosphamide/fludarabine/alemtuzumab/rituximab, fludarabine/cyclophosphamide/obinutuzumab, fludarabine/rituximab/prednisone, fludarabine/rituximab/dexamethasone, fludarabine/alemtuzumab, bendamustine/obinutuzumab, rituximab/cyclophosphamide, rituximab/cyclophosphamide/prednisone, rituximab/cyclophosphamide/dexamethasone, rituximab/cyclophosphamide/vincristine/prednisone, rituximab/cyclophosphamide/vincristine/doxorubicine/prednisone obinutuzumab, ofatumumab, alemtuzumab, nivolumab, idelalisib, idelalisib/rituximab, buparlisib, AT7519M, valproic acid.

The types of treatment ranged from single-agent chemotherapeutics to chemo-immunotherapy and frontline B-cell receptor (BCR)-targeting agents. Until 2014, most treatments were comprised of fludarabine and chlorambucil-containing regimens along with bendamustine and rituximab. Subsequent years saw the introduction of newer agents, including obinutuzumab and ibrutinib. From 2015 on, ibrutinib was being utilized for progressively more patients. Venetoclax was first introduced in 2017 but was reserved for sequential treatment post-ibrutinib with a short follow-up period (Figure 6).

### 3.5. Cause of Death

**Figure 7:**
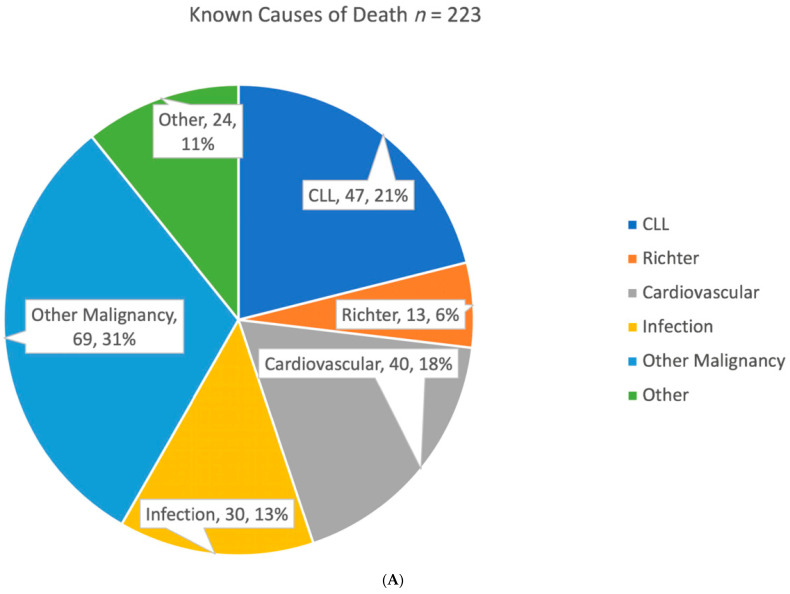

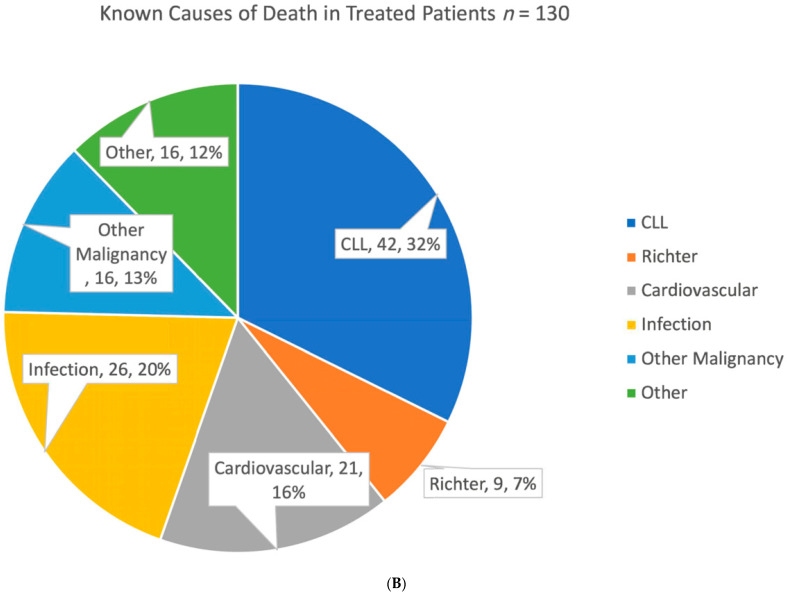

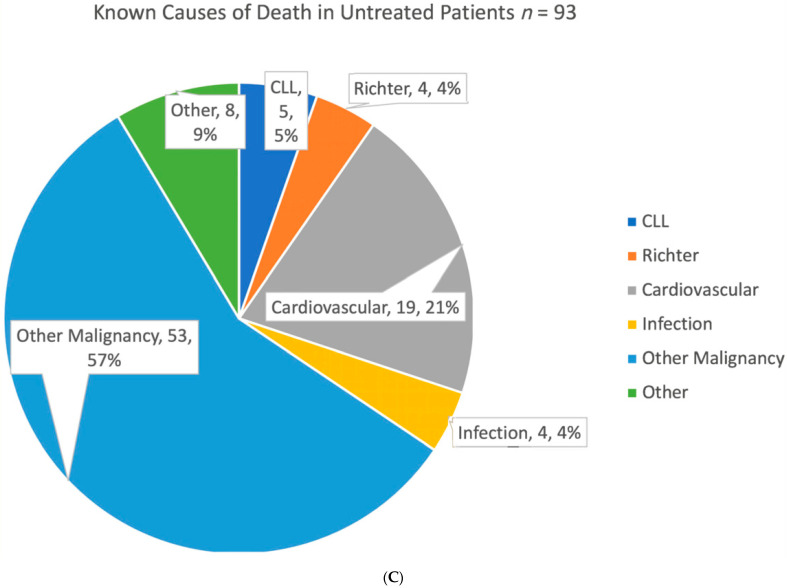
Causes of death. (**A**) Causes of death in the entire cohort (*n* = 233). (**B**) Causes of death in patients who have received treatment (*n* = 130). (**C**) Causes of death in patients who are untreated (*n* = 93). “Other” includes renal failure, sudden death, respiratory failure, old age, collagen vascular disease, multiple comorbidities, generalized deterioration, graft versus host disease, 22 motor vehicle crashes, cirrhosis, Alzheimer’s disease, splenic rupture, venous thromboembolism, multiorgan failure, seizures, pulmonary fibrosis.

380 of 1315 (28.9%) patients died in the study period; 189 (49.7%) of these deaths were in untreated patients, and 191 (50.3%) were in those who received treatment. Causes of death data were available for 223 patients. Amongst treated patients, progression of CLL (*n* = 42, 32%), cardiovascular disease (*n* = 21, 16%), and infection (*n* = 26, 20%) accounted for the majority of deaths. For untreated patients, other malignancies (*n* = 53, 57%) and cardiovascular disease (*n* = 19, 21%) were the largest contributors. Progression of CLL (*n* = 5, 5%) made up a small proportion of deaths in untreated patients (Figure 7).

## 4. Discussion and Conclusions

In the Manitoba centralized CLL clinic between 2006 and 2018, patients were predominantly male, with a median age of diagnosis and median age of treatment similar between the sexes [6,47,48,49]. Most patients were determined to have an early Rai stage (75.9% Rai 0 or I) at diagnosis, likely reflecting access to flow cytometry for diagnosis. CLL-IPI scores were mostly low or intermediate (68.7%) in patients at the time of first treatment. Patients with poorer outcomes were predictably associated with high-risk mutations (i.e., TP53/del(17p), a higher CLL-IPI score, and relapsed disease). Due to the retrospective nature of this study and access to available registry data, only a proportion of patients had known causes of death. Of those identified, the cause of death differed among untreated and treated CLL patients, with secondary malignancies being a major cause of death in untreated patients. It is not entirely clear why this difference exists. One may postulate that the immune dysregulation or cytogenetic aberrations of CLL cells alter their susceptibility to other cancers. Alternatively, it could reflect the increased awareness of screening for second cancers in our province [50,51,52].

The treatment pattern reflected the agents available and those that were standard during the time period. A clear shift occurred in Manitoba when the combination of chlorambucil and obinutuzumab became routinely used after 2014 for the front line, and the availability of subsequent lines of therapy, such as BTK inhibitors, resulted in a decline in fludarabine-based therapies owing to better tolerability and side effect profiles. These changes also increased the availability of tolerable treatments for older adults on the frontlines and all patients in the relapsed setting. Based on our demographics, chlorambucil-obinutuzumab was the most common front-line therapy used, which is reflective of the age and potentially more co-morbid patients associated with a more population-based sample in the province of Manitoba. The use of ibrutinib, or venetoclax, reflects the latest changes to the landscape of CLL therapy in Manitoba. Ibrutinib was found to have efficacy in untreated patients with high-risk molecular features (del17p/TP53 mutation) and IGHV unmutated status in various trials, resulting in improved progression-free survival and OS compared with other conventional therapies [53,54,55]. Revisions to the iwCLL guidelines in 2018 reflected this and recommended its use as a front-line agent in fit patients with unmutated IGHV and those who are del(17p) [28]. Since 2014, ibrutinib has seen widespread use in Manitoba as a second-line agent, and following the iwCLL update, it became a commonplace front-line agent in 2018 for high-risk patients with the del(17p)/TP53 mutation or unmutated IGHV (Figure 6). Venetoclax is in red, BTK inhibitors in yellow, chlorambucil-based therapy in grey, BR/fludarabine-rituximab (FR) in purple, FCR in blue, and other regimens in black. We were able to demonstrate in our retrospective analysis that high-risk patients with unmutated IGHV or TP53 aberrations had improved outcomes with BTK inhibitors at any line of therapy. This finding is key to the justification of the molecular tests that predict improved response with certain therapies. It also enables a choice of effective treatments as we ensure equitable treatment options and accessibility. This is even more important with the implementation of venetoclax in combination with obinutuzumab as first-line therapy with a fixed duration, which continues to shift the treatment landscape [36]. It remains important to acknowledge that the current landscape will need to evolve to respect the personalized differences in treatment based on molecular testing but also with respect to the cost of therapy in a public funding system [56,57]. 

The distribution of cytogenetic abnormalities in our clinic population was similar to Dohner’s report on the genetic landscape of untreated CLL [13]. We observed that patients with high-risk FISH had worse survival in comparison with other risk categories, consistent with expected outcomes [11,17,20]. Since FISH results are known to change over time or with exposure to treatment, it is possible that patients who developed novel high-risk cytogenetic abnormalities or who had a complex karyotype was not captured in this study. The proportion of patients with unmutated IGHV status was in keeping with population studies that suggested 40–50% of patients would have unmutated status [3,19]. We observed that patients who were IGHV unmutated had a nonsignificant trend towards worse OS that is expected from previously published data. However, with the availability of ibrutinib and its benefit in IGHV-unmutated patients, we may no longer see these expected differences [33]. This is similarly observed in patients with del(11q), which prior to ibrutinib was associated with a poorer prognosis [58]. As a result, since April 2018, we routinely perform FISH, IGHV, and TP53 testing prior to the consideration of treatment for patients in Manitoba. While every patient now receives upfront testing prior to consideration of treatment, this was not standard of care throughout the study period and is currently not widely adopted in Canada, which is reflected in our recently published national guidelines [59]. Patients with unmutated IGVH and/or p53 abnormalities represent a population at risk of poor outcomes with chemotherapy, as seen by the OS benefit seen with the introduction of BTKi, which we observed in our centralized clinic [58], thus supporting the role of testing and treating with BTKi.

The CLL-IPI score was calculated at the time of treatment rather than at diagnosis, reflecting practices in a publicly funded health care system. When applied to our local population, there was a statistically worse overall survival in those who had a CLL-IPI score of 4 points or greater in comparison with lower risk categories. Higher-risk groups in CLL-IPI originally described survival from diagnosis but may also predict time to next treatment [41,42]. This is the first instance of its use in a Canadian population study and was enabled by the integration of patient data through the CLL database. This demonstrates the role of routine testing for del(17p), TP53, and IGHV status to identify high-risk patient groups that benefit from alternative therapy such as ibrutinib in keeping with current guidelines [4,59]. As we did not have routine TP53 testing during the time of data collection, we expect that certain patients may be “under-staged” with regards to risk status, but that these numbers are small. The use of CLL-IPI is becoming common practice for entry into clinical trials as a standard of care. This is also an important reason for the implementation of testing. There needs to be consideration of other tests like complex karyotype as a high-risk prognostic factor that is also predictive of response to venetoclax-based therapy and thus may inform the choice of therapy in certain individuals [60]. 

We found that having been treated was not associated with a worse OS in our clinic cohort. This reflects all-cause mortality and may reflect treatment benefits from an immune dysfunction perspective. However, with each subsequent line of therapy, survival was shorter, which is likely due to the identification of the highest-risk patients and the lower likelihood of benefit in more heavily pre-treated patients. It also points to the fact that CLL patients die of progressive CLL in this cohort. Similar to the literature, patients with higher-risk cytogenetics, unmutated IGHV status, or CLL-IPI were more likely to receive treatment [2,12,18]. Causes of death for untreated patients followed in the clinic were largely related to other malignancies and cardiovascular disease and were similar to other available data [61,62,63]. These could be considered long-term immune dysfunctions from the disease [64]. Progression of CLL was the leading cause for patients that received treatment, and infection was the second highest, which reflects the various regimens prescribed for CLL in that era. Rates of transformation to aggressive lymphomas were in keeping with other population data [8]. This data suggests that patients requiring treatment for CLL are more likely to die as a consequence of the disease itself or from complications of CLL-mediated immune suppression such as second cancers and infections, as suggested by Wang et al., where higher-risk patients have a 3-fold risk of dying from CLL and its complications [65]. Second malignancy rates with BTKi are an ongoing concern. How long-term malignancy rates will differ between venetoclax-based versus BTKi therapies as well as previous chemotherapy is yet to be seen. What remains to be seen with novel agents is whether the sequence of treatment—chemotherapy-free, time-limited treatment or continuous novel agents and retreatment without chemotherapy—will improve survival outcomes by eliminating chemotherapy and its long-term sequelae.

In this retrospective study, we described the epidemiology and clinical outcomes of CLL patients in a single centralized Canadian CLL clinic. Our study spans over the course of a decade, capturing a critical shift in the CLL treatment paradigm from chemoimmunotherapy to targeted agents. The unique focus of this clinic allowed us to describe disease aggressiveness from the first and subsequent lines of therapy over time. We demonstrated the validity of mutational analysis and the CLL-IPI score in correlation to survival, as well as the effect of BTK inhibitors on survival in high-risk patients in clinical practice outside of a clinical trial. The cause of death differed significantly among untreated and treated patients, underscoring the importance of immune dysregulation and treatment-related causes of mortality and morbidity.

## Figures and Tables

**Table 1:** Baseline patient characteristics and prognostic features. Proportions are calculated down the first column for the total population and across columns for the remainder of the columns.

	Total	Male	Female	Treated	Untreated
**Number of patients**	1315	791 (60.2%)	524 (39.8%)	530 (40.3%)	785 (59.7%)
**Median age at diagnosis, years**	67.9 (33.6–99.2)	67.4 (33.6–94.1)	68.6 (34.1–99.2)	66.4 (34.1–91.8)	68.7 (36.2–94.1)
**Median age at first-line therapy, years**		69.8 (39.8–97.7)	70.3 (35.2–98.7)	69.9 (35.2–98.7)	
**Malignancy at diagnosis**					
**CLL**	900 (68.4%)	554 (61.6%)	346 (38.4%)	411 (45.7%)	489 (54.3%)
**SLL**	215 (16.3%)	129 (60.0%)	86 (40.0%)	105 (48.8%)	110 (51.2%)
**MBL**	200 (15.2%)	108 (54.0%)	92 (46.0%)	14 (7.0%)	186 (93.0%)
**Rai stage at diagnosis (*n* = 900)**					
**0**	465 (51.7%)	263 (56.5%)	202 (43.4%)	151 (32.5%)	314 (67.5%)
**1**	218 (24.2%)	119 (54.6%)	72 (33.0%)	119 (54.6%)	99 (45.4%)
**2**	73 (8.1%)	48 (65.8%)	25 (34.2%)	43 (58.9%)	30 (41.1%)
**3**	29 (3.2%)	19 (65.5%)	10 (34.5%)	18 (62.1%)	11 (37.9%)
**4**	21 (2.3%)	17 (81.0%)	4 (19.0%)	15 (71.4%)	6 (28.6%)
**Unknown**	94 (10.4%)	60 (63.8%)	34 (36.2%)	66 (70.2%)	28 (29.8%)
**CLL-IPI risk categories (*n* = 233)**	(17.7%)	150 (64.4%)	83 (35.6%)	179 (76.8%)	54 (23.2%)
**Very high (7–10)**	11 (4.7%)	8 (72.7%)	3 (27.3%)	10 (90.9%)	1 (9.1%)
**High (4–6)**	62 (26.6%)	38 (61.3%)	24 (38.7%)	52 (83.9%)	10 (16.1%)
**Intermediate (2–3)**	100 (42.9%)	68 (68.0%)	32 (32.0%)	82 (82.0%)	18 (18.0%)
**Low (0–1)**	60 (25.8%)	36 (60.0%)	24 (40.0%)	35 (58.3%)	25 (41.7%)
**IGHV mutation status (*n* = 835)**					
**Mutated**	482 (57.7%)	279 (57.9%)	203 (42.1%)	160 (33.2%)	322 (66.8%)
**Unmutated**	353 (42.3%)	226 (64.0%)	127 (36.0%)	238 (67.4%)	115 (32.6%)
**Genomic abnormalities by FISH (*n* = 411)**					
**TP53**	5 (1.2%)	3 (60.0%)	2 (40.0%)	5 (100%)	0 (0%)
**Del(17p)**	30 (7.3%)	21 (70.0%)	9 (30.0%)	24 (80.0%)	6 (20.0%)
**Del(11q)**	65 (15.8%)	43 (66.2%)	22 (33.8%)	55 (84.6%)	10 (15.4%)
**Trisomy 12**	94 (22.9%)	61 (64.9%)	33 (35.1%)	74 (78.7%)	20 (21.3%)
**Normal**	96 (23.4%)	54 (56.3%)	42 (43.8%)	66 (68.8%)	30 (31.3%)
**Del(13q)**	187 (45.5%)	119 (63.6%)	68 (36.4%)	134 (71.7%)	53 (28.3%)
**FISH risk categories (*n* = 411)**					
**High**	34 (8.3%)	23 (67.6%)	11 (32.4%)	28 (82.4%)	6 (17.6%)
**Del(11q)**	60 (14.6%)	39 (65.0%)	21 (35.0%)	50 (83.3%)	10 (16.7%)
**Intermediate**	172 (41.8%)	102 (59.3%)	70 (40.7%)	124 (72.1%)	48 (27.9%)
**Low**	128 (31.1%)	82 (64.1%)	46 (35.9%)	88 (68.8%)	40 (31.3%)
Other *	17 (4.1%)	9 (52.9%)	8 (47.1%)	12 (70.6%)	5 (29.4%)

* Includes del(6q) and duplicate 13q. CLL, chronic lymphocytic leukemia; SLL, small lymphocytic lymphoma; MBL, monoclonal B-cell lymphocytosis; CLL-IPI, Chronic Lymphocytic Leukemia-International Prognostic Index; IGHV, immunoglobulin heavy chain variable region gene; FISH, fluorescence in-situ hybridization; Del(17p), deletion 17p; Del(11q), deletion 11q; Del(13q), deletion 13q.

**Table 2:** Median Survival at each line of treatment.

Line of Treatment	Number of Patients	Median Survival (years)	95% Confidence Interval
First-line therapy	530	8.81	6.82–10.79
Second-line therapy	252	5.81	5.16–6.46
Third-line therapy	126	4.69	3.46–5.93
Fourth-line therapy	67	2.97	1.51–4.43

**Table 3:** Time to first and subsequent lines of treatment and types of treatment.

Median Time to First Treatment in Treated Patients (months)	27.0 (0.0–469.0)				
Number treated with	Total	Chemotherapy ^†^	Chemo-immuno-therapy ^‡^	Bruton kinase inhibitors ^§^	Other agents ^||^
First-line therapy	530 (40.3%)	188 (35.5%)	296 (55.8%)	40 (7.5%)	6 (1.1%)
Second-line therapy	252 (19.2%)	58 (23.0%)	123 (48.8%)	52 (20.6%)	19 (7.5%)
Third-line therapy	126 (9.6%)	15 (11.9%)	60 (47.6%)	30 (23.8%)	21 (16.7%)
Fourth-line therapy	67 (5.1%)	3 (4.5%)	21 (31.3%)	25 (37.3%)	18 (26.9%)
Further lines of therapy ^∗^	36 (2.7%)	4 (11.1%)	11 (30.6%)	9 (25.0%)	12 (33.3%)

^∗^ Up to eighth-line therapy. ^†^ Includes: Fludarabine, fludarabine/prednisone, fludarabine/cyclophosphamide, fludarabine/busulfan/methotrexate, cyclophosphamide/prednisone, cyclophosphamide/dexamethasone, cyclophosphamide/vincristine/prednisone, chlorambucil, chlorambucil/prednisone, bendamustine. ^‡^ Includes: fludarabine/rituximab, fludarabine/cyclophosphamide/rituximab, cyclophosphamide/fludarabine/alemtuzumab/rituximab, fludarabine/cyclophosphamide/obinutuzumab, fludarabine/rituximab/prednisone, fludarabine/rituximab/dexamethasone, fludarabine/alemtuzumab, chlorambucil/rituximab, chlorambucil/obinutuzumab, bendamustine/rituximab (+/− idelalisib), bendamustine/obinutuzumab, rituximab/cyclophosphamide, rituximab/cyclophosphamide/prednisone, rituximab/cyclophosphamide/dexamethasone, rituximab/cyclophosphamide/vincristine/prednisone, rituximab/cyclophosphamide/doxorubicin/vincristine/prednisone. ^§^ Includes: Ibrutinib, acalabrutinib, acalabrutinib/obinutuzumab, ^||^ Includes: obinutuzumab, ofatumumab, venetoclax, alemtuzumab, nivolumab, idelalisib, idelalisib/rituximab, buparlisib, AT7519M, valproic acid.

**Table 4:** Time to next treatment (TTNT) from the end of prior line of therapy.

Line of Treatment	Number of Patients	Median TTNT (years)	95% Confidence Interval
First-line therapy	489	3.34	2.71–3.98
Second-line therapy	211	1.90	1.49–2.30
Third-line therapy	105	1.48	0.98–1.98
Fourth-line therapy	50	1.57	0.95–2.20

## Data Availability

For original data, please contact vbanerji@cancercare.mb.ca.
